# Experiences and preferences of truth-telling in families of children with cancer: A phenomenological study

**DOI:** 10.1016/j.apjon.2024.100500

**Published:** 2024-05-03

**Authors:** Yen-Gan Chiou, Shih-Ying Chen, Li-Min Wu, Yea-Ing Lotus Shyu, Yi-Chien Chiang, Chih-Cheng Hsiao, Hui-Chuan Wu, Woung-Ru Tang

**Affiliations:** a Department of Nursing, Kaohsiung Chang Gung Memorial Hospital, Kaohsiung, Taiwan; b School of Nursing, College of Medicine, Chang Gung University, Taoyuan, Taiwan; c School of Nursing, Kaohsiung Medical University, Kaohsiung, Taiwan; d Dementia Center, Department of Neurology, Linkou Chang Gung Memorial Hospital, Taoyuan, Taiwan; e Department of Nursing, Chang Gung University of Science and Technology, Taoyuan, Taiwan; f Division of Hematology/Oncology, Department of Pediatrics, Kaohsiung Chang Gung Memorial Hospital, Kaohsiung, Taiwan; g Department of Post Baccalaureate Nursing, College of Medicine, I-SHOU University, Kaohsiung, Taiwan; h Department of Nephrology, Linkou Chang Gung Memorial Hospital, Taoyuan, Taiwan

**Keywords:** Family caregiver, Truth-telling, Cancer diagnosis, Preferences, School-age children

## Abstract

**Objective:**

The delivery of bad news is an unpleasant but necessary medical procedure. However, few studies have addressed the experiences and preferences of the families of school-aged children with cancer when they are informed of the children's condition. This study aimed to explore families of school-age children with cancer for their preferences and experiences of truth-telling.

**Methods:**

This descriptive phenomenological qualitative research was conducted using focus group interviews and semistructured interview guidelines were adopted for in-depth interviews. Fifteen families participated in the study. The data were analyzed using Colaizzi's analysis. Data were collected from August 2019 to May 2020.

**Results:**

The study identified two major themes: “caught in a dilemma” and “kind and comprehensive team support.” The first major theme focused on families' experiences with cancer truth-telling. Three sub-themes emerged: (1) cultural aspects of cancer disclosure, (2) decision-making regarding informing pediatric patients about their illness, and (3) content of disclosure after weighing the pros and cons. The second major theme, which revealed families’ preferences for delivering bad news, was classified into three sub-themes: (1) have integrity, (2) be realistic, and (3) be supportive.

**Conclusions:**

This study underscores the dilemma encountered by the families of children with cancer after disclosure and their inclination toward receiving comprehensive information and continuous support. Health care personnel must improve their truth-telling ability in order to better address the needs of such families and to provide continuous support throughout the truth-telling process.

## Introduction

Childhood cancer is a critical global public health issue, with approximately 400,000 children diagnosed worldwide each year.^1^^,^^2^ The physical and psychological pressure of a cancer diagnosis on children and their family members is enormous, and it begins immediately after the confirmed diagnosis, when the physician reveals the bad news. Truth-telling is a process of delivering bad news—including the cancer diagnosis and treatment plan—to pediatric cancer patients and their families. A well-conducted cancer truth-telling can reduce family members’ anxiety and promote better doctor–patient communication and collaboration.

However, cultural differences in cancer truth-telling should be recognized by health care personnel (HCP).^3^, ^4^, ^5^ Western countries emphasize patients' autonomy; thus, physicians usually tell patients about their condition directly and frankly. In Eastern countries, family-centered truth-telling culture is valued by HCP,^3^ and physicians may follow the parents’ opinions to hide the truth from the children. Studies in India have shown that up to 68% of Indian parents do not disclose the diagnosis to their ill children as they want to protect them.^6^ In contrast, European countries and the United States advocate freedom and respect for autonomy, and physicians tend to inform the patients directly about their condition, giving children the same right to be informed as their parents.^7^ Children with cancer are informed of their diagnosis and allowed to participate in decision-making.^8^ This highlights the impact of cultural differences on the desire to inform children about their condition.

Although the right of school-aged children to express their opinion and have them considered is emphasized by the convention on the rights of the child implementation, most HCP in Taiwan focus on families' opinions and omit the child's right to know.^9^ An Australian study found that children between the ages of five and 12 with childhood cancer want to be informed about their treatment.^10^ In contrast, studies in China have shown that physicians often assume that family members are the recipients of information and decision-makers for the child. The decision to inform the child depends on the family's attitude^11^^,^^12^ as the family's experience with truth-telling may affect the child's perception of their condition and confidence in the treatment.^13^

Moreover, the child's age and development may influence the family's decision about truth-telling.^14^ Parents are likely to tell their children bad news in person rather than through HCP.^15^ School-aged children aged 6–12 are gradually moving away from self-centeredness and developing the ability to think logically, understand their illness, and make thoughtful decisions.^16^^,^^17^ Studies have shown that they have greater anxiety and fear of hospitalization than younger children.^18^^,^^19^ Therefore, family members of school-aged children may bear more stress from their children, which also affects their experiences of the truth-telling process.

Patient-centered communication based on their preferences can reduce emotional distress, increase trust in medical care, and improve compliance with treatment.^11^^,^^20^, ^21^, ^22^, ^23^ However, inadequate communication during truth-telling can lead to regret and distrust in medical treatment.^24^^,^^25^ Communication skills training (CST) for truth-telling is essential. Nonetheless, most CST programs are designed for adult patients with cancer, and CST tailored for families and pediatric patients with cancer is lacking. Therefore, this study explored the truth-telling preferences and experiences of family members of school-aged children with cancer, aiming to provide some insight into CST for oncology nurses in Taiwan.

## Methods

### Design

This study adopted descriptive phenomenology, which was proposed by Husserl (1859–1938). He suggested that the experience perceived by human consciousness has value and that all assumptions of phenomenology are related to consciousness.^26^^,^^27^ Descriptive phenomenology is the best choice when the research topic intends to gain insight into the meaning of experience in a particular case.^28^^,^^29^ Data were collected from August 2019 to May 2020.

### Participants and setting

This study used purposive sampling. To increase the credibility of the study, a variety of respondents were selected from among the volunteers via maximum variation sampling, which is a type of purposeful sampling design.^30^ Maximum variation sampling selects participants based on a wide range of variations in backgrounds,^30^ which encompass different sexes, ages, identities, and educational levels. The inclusion criteria were as follows: (1) primary caregivers, who are family members of school-age children (aged 6–12) diagnosed with and receiving treatment for acute lymphoblastic leukemia; (2) individuals who can communicate in Mandarin; and (3) those who agree to participate in this study. They were recruited from the pediatric cancer wards of two medical centers. The attending physicians introduced the family members to the researcher (YGC). The researcher explained the purpose and process of this study to the potential participants and exchanged the contact information (email and phone number) with family caregivers to further arrange the focus group if they were willing to participate in the study. The focus group interviews were conducted after participants had completed the written informed consent form. All the potential participants agreed to participate in this study.

### Data collection

This study used focus group interviews to harness dynamic interactions among the participants, thus enabling them to build upon each other's responses and generate ideas.^31^ A well-conducted focus group fosters trust among participants, facilitating the sharing of diverse experiences and emotions. The researcher (YGC), a nursing PhD candidate who has been trained in qualitative research, conducted all the interviews as a moderator. Another researcher (WRT) also participated in the interviews, acting as an assistant moderator in order to coordinate technologies, take notes, and observe the behaviors of the participants.^31^ At the beginning of the interview, the researcher (YGC) introduced herself and provided a brief introduction to the study aim in order to evoke parents' interests and to establish a relationship. The researcher conducted three focus group interviews, and each group consisted of five parents.

A nursing professor (WRT) and two nursing PhD candidates (YGC and SYC) trained in qualitative research designed the semistructured interview guide, which was based on the existing literature^17^^,^^32^ and suggestions from pediatric oncology experts (CCH and YCC). A semistructured interview guide was used to obtain rich, informative, and multiperspective interview data. Six interview questions were asked: (1) can you tell us about a recent experience of being told about your child's condition? (2) How do you think HCP should tell parents in your situation that their child has cancer? (3) How should HCP tell a sick child about their condition and treatment? (4) Do you think HCP should tell you and your child everything about the child's condition, or do you think some conditions do not need to be told? (5) How do you think the HCP should tell you and the child that the treatment is no longer effective? (6) Would you like to share anything that is relevant to telling the truth to the child regarding their condition?

The participant was the primary caregiver of the sick child, who knew the child's condition the most. A total of 15 participants were included in this study. The researcher (YGC) bracketed prior experiences to reduce personal bias, then acted as a focus group facilitator by asking questions and encouraging the participants to express their opinions and share their experiences. Data saturation was achieved when the researcher could accurately predict the family's response to each question or when the collected data were repetitive without providing new information, codes, or themes.^33^ After this, no new participants were recruited. The data saturation was assessed by both the moderator (YGC) and the assistant moderator (WRT) in order to avoid researcher bias. The focus groups lasted for 100–114 minutes, averaging 108 minutes. The verbatim transcription was completed and checked within 48 hours after the focus group. A gift voucher of NTD 200 was provided to each participant upon completion of the interview.

### Data analysis

Two researchers (YGC and WRT) conducted data analysis independently by using the descriptive phenomenology approach proposed by Colaizzi.^34^ Any disagreements were solved through discussion and by consulting the viewpoint of a third researcher (SYC) in order to obtain consistency, thus resulting in full consensus. The seven steps of the analysis were as follows: (1) reading the descriptions of the research participants and becoming familiar with the content; (2) extracting phrases or sentences from the transcript directly related to the phenomenon under investigation (significant statements); (3) piecing together the meaning of each significant statement (meaning); (4) organizing the aggregated meanings into clusters of themes: examining whether the original meanings included elements; (5) integrating the findings into a detailed description of the explored themes; (6) identifying the basic structure and describing the phenomenon under investigation; and (7) completing the final validation step by returning to each theme. This final step was achieved by asking the following questions to the participants: “How do the findings of my description compare with your experience?” Any relevant new information that emerged during the process was incorporated into the findings of this study. In this study, no relevant new information emerged during this step.

### Rigor and trustworthiness

This study's rigor—including the findings' of credibility, transferability, dependability, and confirmability—was evaluated by the concept of trustworthiness, as proposed by Lincoln and Guba (1985).^35^ The researcher (YGC) has more than 25 years of experience in cancer care. Audio recordings of all focus groups were created and transcribed verbatim within 48 hours after the interviews were completed. Data analysis was conducted after the consistency between the word-for-word manuscript and the audio recordings was confirmed by another researcher (HCW). The impact of single institutional and regional factors on the findings was reduced by including participants from two medical centers in southern and northern Taiwan, and the conversations and observations of the focus groups were described robustly. This study was also peer-reviewed by a nursing professor (WRT) with experience in qualitative research who randomly selected one-sixth of the transcripts and coded them independently and simultaneously with the researcher (YGC). The percentage of the same codes was calculated manually, representing the consistency of the analyses; this consistency was 88%, indicating the dependability of the study content. Finally, this study's rigor was ensured by asking for the participants' feedback to verify whether the findings aligned with their experiences and perceptions. Additionally, the descriptive phenomenology approach was coached and confirmed by two experts in qualitative research (YIS and YCC).

### Ethical considerations

This study was approved by the Institutional Review Board (IRB No. 201900508B0). The researcher (YGC) explained the purpose and methodology of the study to the participants before proceeding with the study. Participants were also allowed to withdraw from the study at any time. The participants’ names were replaced with codes. Moreover, any private information was kept confidential and not made publicly available. All of the obtained data were used solely for academic purposes. The interviews were conducted only after consent forms were signed by the participants.

## Results

A total of 15 family members were included in this study, including 12 women (80.0%) and three men (20.0%). Excluding the three grandparents, the average age of the participants’ remaining 12 family members was 41 years old; 12 were married (80.0%), and three were single (20.0%). Most participants had a senior high school education or above (93.3%). All children had been diagnosed with acute lymphoblastic leukemia, with only one of them having had a cancer relapse; the rest of the children had been diagnosed with the disease for the first time (Table 1). The study identified two major themes: “caught in a dilemma” and “kind and comprehensive team support.”

**Table 1:** Participants’ characteristics (*N* = 15).

No.	Identity	Age (year)	Gender	Educational level	Marital status	Children's age (year)	Children's gender
P1	Mother	44	F	Junior college	Married	10	F
P2	Mother	40	F	Junior college	Married	9	F
P3	Grandmother	57	F	Senior high school	Married	7	M
P4	Father	47	M	Senior high school	Married	9	M
P5	Mother	37	F	Senior high school	Divorced	9	M
P6	Mother	43	F	Junior college	Married	11	M
P7	Mother	43	F	Junior college	Married	8	F
P8	Grandfather	60	M	Junior high school	Married	8	M
P9	Mother	38	F	Junior college	Married	7	M
P10	Mother	40	F	University	Widowed	10	M
P11	Mother	41	F	University	Married	6	M
P12	Mother	38	F	Senior high school	Married	7	M
P13	Grandfather	73	M	Senior high school	Married	10	M
P14	Mother	50	F	Senior high school	Divorced	10	M
P15	Mother	38	F	Senior high school	Married	6	M

F, female; M, male.

The major theme “caught in a dilemma” had three sub-themes: cultural aspects of disclosure in Chinese society, decision-making regarding informing pediatric patients about their illness, and the content of such disclosure after weighing the pros and cons. As for the other major theme “kind and comprehensive team support,” it had three sub-themes: have integrity, be realistic, and be supportive (Table 2).

**Table 2:** Sub-themes and theme clusters of two themes.

Themes	Sub-themes	Theme clusters
Caught in a dilemma	Cultural aspects of disclosure in Chinese society	Direct disclosure to family members by HCP
		Disclosure to pediatric patients using metaphorical methods by HCP
	Decision-making regarding informing pediatric patients about their illness	Facilitators
		Barriers
	The content of disclosure after weighing the pros and cons	Age and level of understanding
		The content of the disclosure regarding the child's condition
Kind and comprehensive team support	Have integrity	Honesty in disclosure
		Patience in answering questions
	Be realistic	Information on disease management
		Information on daily care
		Pessimistic prognosis
	Be supportive	Importance of psychological care
		Sources of support

HCP, health care personnel.

### Theme 1: caught in a dilemma

In Chinese culture, the primary caregivers of children with cancer are typically the ones who receive the disclosure about their diagnosis. They are responsible for coping with the immediate impact of hospitalization and treatment while also considering the child's age. They also have to navigate the dilemma of whether—and how—to disclose the illness to the child.

#### Sub-theme 1: cultural aspects of disclosure in Chinese society

Chinese people represent a substantial proportion of the population in many Asian countries, and in Chinese society, minors are considered a vulnerable group without autonomy. Medical decisions are typically made by parents or legal guardians, and HCPs primarily communicate information about the patient's condition to the parents or primary caregivers. This sub-theme encompasses two theme clusters: direct disclosure to family members by HCP and disclosure to pediatric patients using metaphorical methods by HCP.

##### Theme cluster 1: direct disclosure to family members by healthcare personnel

Although the disclosure of illness includes information about diagnosis, treatment, side effects, and prognosis, what impresses family members most is the “diagnosis disclosure.” This is because the family is not psychologically prepared for their child to have cancer, let alone long-term hospitalization. The moment in which the physician directly informs them of the diagnosis is unforgettable for them. A family member recalled being informed by a physician during a visit to the outpatient clinic:

*We were at the clinic to see the blood test results, and that was when we found out that she seemed to have leukemia. The doctor said she had to be hospitalized immediately and undergo treatment for a month. We found out about it under those circumstances. (P2: 40-year-old mother)*

Another family member brought their child to the hospital for an urgent check-up due to many abnormal bruising spots on the child's body. Although it had happened a year before, the impact on the family at the time was considerable due to the physician's direct disclosure of the diagnosis. The family member vividly described what the physician had said:

*I remember it very clearly. The doctor said to me after the examination results came out, “Grandma, your child’s bruising spot (under the clavicle) is a typical presentation of leukemia, and the leukemia index is 58,000.” I remember it very clearly! (P3: 57-year-old grandmother)*

##### Theme cluster 2: disclosure to pediatric patients using metaphorical methods by healthcare personnel

Most physicians disclose the diagnosis directly to family members. However, for a few curious pediatric patients, when informing them, physicians commonly use indirect or metaphorical ways of making the disclosure, such as bacteria or insects. A family member described the way in which the physician informed the child of his condition:

*The doctor told him (the pediatric patient) that he was sick because there were bad bacteria or bugs in his body, and now the doctor would remove those bugs. (P10: 40-year-old mother)*

#### Sub-theme 2: decision-making regarding informing pediatric patients about their illness

According to the Taiwan Pediatric Oncology Group (TPOG) standard protocol, acute lymphoblastic leukemia requires immediate hospitalization for 4–6 weeks of induction therapy during the acute phase, and the entire treatment course takes at least 2–3 years to complete. Family members immediately face the decision of whether to tell the truth to the pediatric patient. This sub-theme included two theme clusters: facilitators and barriers.

##### Theme cluster 1: facilitators

Fifty percent of family members believe that disclosing the child's condition has many benefits in clinical practice as, during the chemotherapy process, the child needs to undergo many invasive procedures, such as lumbar puncture, placement of a port-a-cath, and intravenous injection of chemical drugs. If the children understand their conditions and the importance of treatment, it can increase medical compliance.

*We would tell her, “If you don’t get the shot, you will have to stay in the hospital, and if you don’t cooperate with the doctor’s treatment, your body will become unhealthy, and you will become more and more uncomfortable, or it will make you suffer in some way.” It feels a bit intimidating to her. (P2: 40-year-old mother)*

Due to the side effects of chemotherapy drugs, pediatric patients’ immune function and resistance can be reduced. Physicians therefore ask children to wear masks when going out and prohibit them from eating raw food; to comply, children need to fully understand these requirements. Family members believe that it is necessary to tell children the truth to ensure treatment safety and let children understand the consequences of noncooperation. One father said,

*I have told my child that it’s okay, he knows what disease he has and what food he cannot eat. This makes treatment safer. When the air is bad, you have to wear a mask when going out. He needs to know how to wear a mask and take basic precautions, so he won’t be afraid. (P4: 47-year-old father)*

##### Theme cluster 2: barriers

The fear of frightening the child is the main reason why family members are hesitant to inform pediatric patients of their condition. Additionally, they may not be able to predict the child's reaction to hearing about the cancer diagnosis, or they may be unsure of how to answer the child's questions directly. These worries make it difficult for them to disclose the truth. One family member said,

*We did not inform the child because I think the child's first reaction would be, “What will happen to me? Will it hurt? Will I have to take more medicine? Will I have more injections?” Adults tend to consider the situation more comprehensively. I will discuss what we can do next with the father. (P2: 40-year-old mother)*

Some family members are concerned that children may associate cancer with death, which could influence their willingness to disclose the truth, as children over the age of 9 years know that death results in the end of life and disappearance of the body. Some children may have even experienced the death of an older family member and may be afraid of death. Therefore, family members may avoid using the term “cancer” when informing them about the illness.

*I dare not say to him “you have leukemia” because he associates the term “cancer” with death. He attended the funeral of his aunt, who died of cancer, and he knows that it can be fatal. He would be scared. Therefore, we avoid using the word “cancer” and instead use “illness” when speaking with him. (P3: 57-year-old grandmother)*

#### Sub-theme 3: the content of disclosure after weighing the pros and cons

The primary consideration for family members when deciding whether to disclose the disease to the children is their age. During the school-age period, which is between six and 12 years old, children develop from being illiterate to being able to recognize and use most words. Family members believe that they should first assess the child's level of understanding of the disease before informing them, and most believe that it is not suitable to inform young children about their illness. However, for older children, it may be appropriate to inform them and discuss their condition with them. Two themes were identified from the data analysis: age and level of understanding, and the content of disclosing the child's condition. Two theme clusters were included as follows:

##### Theme cluster 1: age and level of understanding

Leukemia is more common in children aged 2–6 years old—a stage of life when they are still developing their understanding of the world. Parents must explain to their sick children why they need to be hospitalized and undergo long and intensive treatment, and failing to provide an explanation may lead to the child's imagination and misunderstanding of their situation. However, how to explain, what to say, and how much to disclose are critical considerations. The most important aspect is whether the child can accept and cooperate with treatment after being informed of their condition. One family member stated,

*This depends on the age of the child when they become ill. At the age of 9, they may be able to understand, but sometimes children aged 3–5 cannot really comprehend. It depends on their age! That’s what I think. (P7: 43-year-old mother)*

Family members believe that older children have a relatively greater understanding of language than younger children and can easily obtain disease-related information through the internet or media. They can comprehend the situation; thus, family members tend to disclose the truth to them.

*Because he is older, he was in the fourth grade (10 years old) when he became ill, and now he is in the sixth grade (12 years old). That’s it! I think he can understand this. (P6: 43-year-old mother)*

##### Theme cluster 2: the content of the disclosure regarding the child's condition

When a child becomes ill at a young age, they may not be able to comprehend why they are sick and need to be hospitalized; therefore, family members must explain this in a way that the child can understand. A parent with a nursing background used the metaphor of having worms in the body to explain the illness to the child, believing that the child could accept it.

*Because the child was very young when he became ill, he actually didn’t understand anything. I didn’t tell him about the diagnosis. I just explained to him that there were many bugs in his body biting him … which prevented him from walking, and he was able to accept it at that time. (P12: 38-year-old mother)*

### Theme 2: kind and comprehensive team support

Family caregivers who are responsible for taking care of children with cancer have certain preferences with regard to the disclosure of the disease. These preferences indicate their comprehensive needs. They require healthcare teams to be honest and friendly, provide detailed explanations of the complete treatment plan, and offer continuous care and support to family caregivers.

#### Sub-theme 1: have integrity

This sub-theme reflects the requirements of family members on the attitude of physicians when disclosing the disease. In this study, 93.3% of children were diagnosed with cancer for the first time, and after the disclosure of disease status by physicians, their family members expressed expectations and demands for better attitudes from physicians in future disclosures. This includes two theme clusters: honesty in disclosure and patience in answering questions.

##### Theme cluster 1: honesty in disclosure

Honest and frank disclosure of disease status by physicians can alleviate the uncertainty that family members may feel about the future and reduce their anxiety toward treatment. Faced with the unequal distribution of medical information, family members hope to receive truthful disease prognosis information, as they require sufficient knowledge to face the challenges of the disease.

*Doctors must be honest. If the family members do not know, what if the child suddenly passes away? Unlike adults, some of whom may accept their diagnosis and limited lifespan, children do not understand and may complain about why they have to suffer from this disease. (P3: 57-year-old grandmother)*

##### Theme cluster 2: patience in answering questions

The literature has suggested that family members may experience information overload and emotional distress when physicians disclose the disease status; they may not fully comprehend or digest the information provided by physicians at the time of disclosure. Therefore, repeated explanations and reserved time are necessary to allow family members to ask questions and receive answers. One family member described her attending physician in the following way:

*One of the physicians would repeatedly explain until you understood when you asked him questions. He was patient and never felt annoyed. (P11: 41-year-old mother)*

#### Sub-theme 2: be realistic

This sub-theme describes the scope of information that family members hope to receive from physicians during the disclosure of disease status. In addition to a complete and detailed treatment plan and information on disease management, family members also hope to receive information on daily care to better take care of the sick child. Furthermore, if the treatment is ineffective, family members hope that physicians will inform them in advance so that they can prepare accordingly. This included three theme clusters: information on disease management, information on daily care, and pessimistic prognosis.

##### Theme cluster 1: information on disease management

When a child is diagnosed with cancer, physicians inform the parents of the diagnosis and treatment. Family members expressed a great need to understand leukemia and its complete and detailed treatment plan through professionals. Although they may obtain disease information from different sources, they often, cannot distinguish the truthfulness of the information and therefore feel confused. One family member stated

*I really want to know the side effects of the injection (Oncovin), the side effects of the oral medication (cortisone), and the side effects of the others, as we see few side effects written on the medicine bags, but the information found on the Internet is very scary. (P3: 57-year-old grandmother)*

Another family member expressed a desire for physicians to directly explain how to treat the child's illness. For them, knowing the treatment plan and outcome are the most important information; thus, physicians (or HCPs, including nurses), should provide a more detailed plan and may have to use written materials to support the provided information.

*The key is how to treat it, that's the key! I think it doesn't matter how you talk about this disease as no one understands it! Just speak directly and provide family members with knowledge in this area, that's all. (P11: 41-year-old mother)*

##### Theme cluster 2: information on daily care

The treatment process for leukemia is rather lengthy and causes severe pain to pediatric patients. Although chemotherapy can kill cancer cells, it can also damage normal cells. Family members believe that it is necessary to make the child understand treatment side-effects and the importance of observing dietary restrictions and activity precautions in daily life. Hospitals should provide relevant information on daily care and activities to assist children in self-care.

*If there is health education (materials) similar to this, that tells you what disease you have, what you will face in the future, what causes it, what to pay attention to in diet, and how to take care of yourself during treatment, I think it is necessary. (P15: 38-year-old mother)*

##### Theme cluster 3: pessimistic prognosis

When asked about their thoughts on whether the child's treatment did not meet expectations, almost all family members remained silent and had a worried expression, indicating that they were not aware that the child could possibly die due to treatment failure. Family members believed that physicians should not let the child know the bad news of treatment failure but should inform the family members first. It is up to the family members to decide the appropriate time and person to inform the child of their condition.

*... (silence for about 10–15 seconds) I think … it’s better for the parents to know first, so that we can tell him more gently. If it were me, I would want to let the parents know first. We will discuss it and then decide who—whether the father or mother or someone else—should tell him. That way, he will be more likely to accept it. (P9: 38-year-old mother)*

#### Sub-theme 3: be supportive

This sub-theme reflected the psychological needs of family members after physicians informed them of the child's condition. Both the child and family members may be under immense pressure and in need of an outlet. The medical team must pay attention to the psychological care of family members and provide sufficient support to help them overcome the difficult times. This included two theme clusters: the importance of psychological care and sources of support.

##### Theme cluster 1: importance of psychological care

For school-aged children with cancer, the long treatment process may take them through multiple stages of social development, from pre-school or school age to the teenage years. The experience of illness may impact their level of mental maturity and perspective on the world. HCP must assist family members in identifying changes in the child and provide psychological support. One worried mother expressed,

*I think the psychological aspect is very important. For example, we are facing her slowly changing personality, and when she is under great psychological pressure and has no way to vent, we do not know who to seek help from. I believe that more attention should be paid to the emotional aspect of these children with cancer. (P2: 40-year-old mother)*

##### Theme cluster 2: sources of support

During the long process of accompanying the child's treatment, family members may inevitably experience moments of low mood and depression, especially when the child's treatment is not going well; they must endure immense pressure and pain that outsiders cannot understand. Therefore, while their child is hospitalized, most family members seek emotional support from other families with similar experiences. One family member stated,

*When I feel a bit blue, I talk to her (another family member of a child with cancer), and I also care about her child. I think this mother is great, and she basically encourages me. We don't talk to outsiders about these things—we just find someone in a similar situation to share our feelings with when we are feeling unhappy. (P1: 44-year-old mother)*

Due to the busy medical environment in Taiwan, physicians come and go in a hurry, and the time spent visiting patients and their families is brief. Therefore, family members seek care and psychological support from other members of the medical team, such as psychologists, nurse practitioners, and clinical nurses to help them overcome difficulties.

*When the psychologist and specialized nurse come together to provide counseling for the child, they may also take notice of my condition and provide me with counseling as well. So, with the two of them, one counseling the child in the ward and the other counseling me outside, I found that the psychologist and specialized nurse were helpful to us. (P1: 44-year-old mother)*

## Discussion

This study is the first in an Asian country to explore the experiences and preferences of family members of school-aged children with cancer regarding disease disclosure. The first major theme, “caught in a dilemma” reflected families' experiences during truth-telling. Three sub-themes were identified: cultural aspects of disclosure, decision-making regarding informing pediatric patients about their illness, and the content of disclosure after weighing the pros and cons. The second major theme, “kind and comprehensive team support” revealed families’ preferences for delivering bad news. Three sub-themes were identified: have integrity, be realistic, and be supportive.

### Experiences and preferences of truth-telling in family members

Under Confucianism, parents have a responsibility to protect their young children, which has a profound influence on Chinese societies such as those in China, Singapore, Korea, and Malaysia. In this cultural context, based on the rationale of protection, parents filter information and decide what should and should not be disclosed to their children. They do this to avoid imposing the negative impacts of the illness and treatment on their children.^11^^,^^21^^,^^36^ In this study, the main receivers of truth-telling who received it directly from the physicians were family members; they then decided whether to disclose the diagnosis to the child, which is consistent with the medical culture, beliefs, and traditions in Taiwan. However, this differs greatly from the cultural practices in North America and European countries, where physicians clearly communicate the patient's condition to the patient.^37^ In addition, although scholars consider disease disclosure to be a process,^5^ when discussing their experiences with disease disclosure, most family members in this study focused on the initial disclosure of diagnosis; this highlights the impact and importance of the physician's first disclosure. In this study, most family members were informed of their child's illness in outpatient clinics or emergency rooms, and the time for disclosure may have been as short as 10–15 minutes or even shorter. Due to the busy medical environment and heavy patient care workload in Taiwan, it is difficult for physicians to consider appropriate arrangements for the disclosure of a cancer diagnosis and to have empathetic conversations.^38^ A scoping review^15^ indicated that most parents wanted accurate information about their child's cancer and needed sufficient time for consultation. The time required for delivering bad news was at least 30–90 minutes, which differs from the experiences of disease disclosure obtained in this study. If disease disclosure can meet the needs of family members, it can reduce many unnecessary troubles and mistrust in the future. HCP must make appropriate time preparations to ensure that family members receive good disease disclosure experiences, thus helping them to make the best clinical decisions.

In this study, most of the family members were “caught in a dilemma” regarding whether to disclose the truth to the child. Similar to the research results on Chinese parents of only children and those on American children aged 7–17, family members were reluctant to expose children to the impact of a serious illness and worried about their fear and anxiety if they knew the truth.^21^^,^^39^ However, they also needed to explain the reason for the illness to facilitate cooperation in the treatment plan. Family members hesitated and faced a dilemma about whether and how to disclose the truth to the child. The research results in various countries suggest that age is the main factor affecting how much information parents transmit to their children. A scoping review showed that parents preferred to deliver bad news to young children themselves, and the younger the children, the less information they were told.^15^ A study in the Netherlands^40^ showed that older children received more diagnosis and prognosis information in the initial stages. Even within Asian countries, the way family members disclose cancer diagnosis to their children varies due to different social and populist influences. A study of children with cancer aged 30 days to 18 years in India^6^ found that only 32% of family members would disclose the diagnosis to their children. The reason was that 54% of the children were under 8 years of age, and family members were concerned that the children were too young to understand; this influenced their unwillingness for disclosure. Another study suggested that the traditional paternalistic model of the doctor–patient relationship in Indian society makes most parents afraid to openly discuss illness and ask questions. Their sense of shame about cancer also made the parents believe that talking about cancer with their children means misfortune; therefore, they tend to withhold information about the illness.^41^ However, a study of children under 18 years of age with cancer in Hong Kong^42^ yielded different results. Parents were more willing to tell their sick children the truth, as the children had the right to know about their illness. The children were informed about the need for long-term treatment and the details of daily life that required attention, taking on the responsibility of self-care. This may be due to Hong Kong's past as a British colony, with a cultural mindset more inclined toward Western countries and a generally accepted value of respecting patients' autonomy internationally. In this study, although family members would use indirect or metaphorical ways to disclose the diagnosis when a child expressed a desire to know about their illness, they would still filter the amount of information the child received to protect them—especially younger children. In the future, any HCP who cares for children with cancer should receive communication skills training for disclosing the diagnosis and reducing the dilemma faced by family members when informing sick children about their condition.

Furthermore, family members hope for “kind and comprehensive team support” that reflects integrity, reality, and support. This was consistent with the findings from other studies.^6^^,^^24^^,^^37^^,^^42^^,^^43^ The results from a study of parents of children with cancer in Africa^44^ showed that 30% of the families did not understand the information provided by physicians, which was attributed to their low levels of education. In addition, scholars^39^ have pointed out that families may feel shocked and stressed by cancer diagnoses, which makes it difficult for them to listen to and digest all the information provided by physicians. Therefore, during the disclosure process, physicians should patiently repeat the information and ask family members to repeat it back to confirm their understanding and avoid anxiety and fear caused by misunderstanding.

Due to differences in cultural backgrounds, patients or family members may have different preferences for receiving bad news. A systematic review^45^ has shown that, compared to Western patients, Asian patients are less likely to discuss life expectancy. The lower tendency to discuss life expectancy among cancer patients in Taiwan is attributed to patients not expressing preferences for prognostic information or HCP not disclosing such information to patients,^46^ which is inconsistent with the findings in this study. In this study, family members expressed a desire for physicians to disclose life expectancy, which may be related to the fact that 80% of the family members participating in this study were women. Research by Taiwanese scholars has shown that women prefer physicians to disclose life expectancy information compared to men.^47^ When the researchers mentioned the disclosure of poor prognosis or ineffective treatment of pediatric patients, all family members remained silent for a period of time before expressing their thoughts. They showed obvious emotional changes in their reactions, which might be due to the fact that most pediatric patients received a cancer diagnosis for the first time, and their families did not believe that their child might enter the terminal stage. This highlights how heavy the burden of a poor prognosis is for family members. Although the quality of end-of-life care in Taiwan ranks third in the world,^48^ the disclosure of prognoses for pediatric cancer patients still needs to be addressed and improved. HCP must continue to receive communication skills training in this field and deliver bad news according to the preferences of each family.

Due to the enormous pressure faced by pediatric cancer patients and their families, family members emphasized the importance of “kind and comprehensive team support,” which was consistent with previous studies.^37^^,^^45^^,^^49^ In contrast to these findings, in the busy medical environment of Taiwan, physicians may find it difficult to provide family members with reassurance and emotional support. Therefore, family members may seek care and emotional support from other channels, such as other patients' families or members of the healthcare team. For example, interaction with nurse practitioners, psychologists, and clinical nurses can assist family members in adjusting their emotions and coping with stress to overcome difficulties. This result highlights the complementary functions of the members within the pediatric cancer care team and the importance of mutual support among patients' families. A study conducted in Hong Kong^42^ also yielded similar results. It is recommended that the healthcare team proactively introduces successful cases and their families to new families of children with cancer during the early stages of hospitalization; this can help form a support group between new and old patients’ families, enabling them to share their care experiences and emotional adjustments.

### Implications for nursing practice and research

This study revealed certain differences between the actual experiences of families of children with cancer with regard to disease disclosure and their preferences. In addition to the busy medical environment, inadequate communication skills training for HCPs may also be a contributing factor. Fujimori^43^ proposed four important aspects of cancer disclosure, including establishing a supportive environment (supportive environment), delivering bad news according to patients' preferences (How to deliver bad news), provision of additional information (additional information), and provision of emotional support (reassurance and emotional support). According to Fujimori et al. reassurance and emotional support form the most important aspects when HCP delivers the bad news;^43^ this echoed theme 2 “kind and comprehensive team support,” which we found in our study. A study in the Netherlands^20^ indicated that a single disclosure of illness cannot satisfy the parents' long-term needs. Physicians must arrange follow-up meetings, which highlights that illness disclosure is an ongoing process. As both the pediatric patients and their parents may have different needs and psychological issues during the lengthy treatment, the need for “kind and comprehensive team support” may vary with the child's age and the stage of the disease. The pediatric cancer care team should receive communication skills training and provide more social and psychological care and support to families and patients to address these differences. It is noteworthy that Taiwan still has considerable room for improvement in creating a supportive disclosure environment. It is necessary to establish a policy of building a friendly healthcare environment through the government health department and improve it by incorporating it into hospital evaluation indicators, thereby meeting families' needs with regard to the disclosure environment.

### Limitations

To the best of our knowledge, this is the first study in Taiwan to explore the experiences and preferences of family members of school-age cancer patients regarding the disclosure of disease. However, this study has three limitations. First, most family members who participated in this study were women, and previous research has confirmed that gender differences can influence perspectives on truth disclosure to patients.^47^ Second, the experiences of grandparents may differ from those of parents. It is evident that the stress borne by a grandmother caring for a sick child appears to be greater than that of a parent. Grandparents rarely receive evidence-based psychosocial support.^50^ Therefore, future research should consider including fathers and grandparents in the sample in order to gain a more comprehensive understanding of the abovementioned topics. Third, family members' views on truth disclosure may be influenced by the timing of the diagnosis. Sisk et al.’s study^51^ indicated that 36% of family members experienced fluctuating feelings in the period between the time of the disclosure and four months later. Moreover, 32% of them are also experiencing changes in preferences between four and 12 months. Future research could focus on recruiting patients diagnosed within one month to minimize recall bias and differences over time. Finally, this study primarily focused on family members of children with leukemia; future research could include children with other types of pediatric cancers to increase the transferability of research results.

## Conclusions

This study investigated the experiences and preferences of the family members of school-aged children with cancer with regard to physicians’ disclosures of the disease. The results identified two major themes: “caught in a dilemma” and “kind and comprehensive team support.” This study underscores the dilemma encountered by the families of children with cancer following disclosure and their inclination toward receiving comprehensive information and continuous support. HCPs must improve their truth-telling ability in order to better address the needs of families and to provide continuous support throughout the truth-telling process. It is essential to ensure that the families of children with cancer receive better quality care throughout the arduous treatment journey.

## Ethics statement

This study was approved by the Institutional Review Board (IRB No. 201900508B0). All participants provided written informed consent.

## Funding

This study was supported by a grant from the Ministry of Science and Technology (Grant No. MOST 108-2511-H-182-005-MY2) matching funding from Chang Gung University (Grant No. BMRP815), and Chang Gung Memorial Hospital (Grant No. CMRPD1K0521). The funders had no role in considering the study design or in the collection, analysis, interpretation of data, writing of the report, or decision to submit the article for publication.

## CRediT authorship contribution statement

**Yen-Gan Chiou:** Conceptualization, Data curation, Formal Analysis, Investigation, Methodology, Resources, Writing – original draft. **Shih-Ying Chen**: Conceptualization, Data curation, Methodology, Writing – original draft. **Li-Min Wu:** Conceptualization, Project administration, Supervision. **Yea-Ing Lotus Shyu**: Conceptualization, Methodology, Project administration, Supervision. **Yi-Chien Chiang**: Conceptualization, Project administration, Supervision. **Chih-Cheng Hsiao**: Conceptualization, Project administration, Resources, Supervision. **Hui-Chuan Wu**: Conceptualization, Formal Analysis. **Woung-Ru Tang**: Conceptualization, Formal Analysis, Funding acquisition, Investigation, Project administration, Resources, Supervision, Writing – original draft. All authors had full access to all the data in the study, and the corresponding author had final responsibility for the decision to submit for publication. The corresponding author attests that all listed authors meet authorship criteria and that no others meeting the criteria have been omitted.

## Declaration of competing interest

The authors declare no conflict of interest. The corresponding author, Professor Woung-Ru Tang, serves as a member of the editorial board of the *Asia-Pacific Journal of Oncology Nursing*. The article has undergone the journal's standard publication procedures.

## Acknowledgments

We thank family caregivers and cancer patients who participated in this project.

## Data availability statement

The data that support the findings of this study are available on request from the corresponding author, Woung-Ru Tang. The data are not publicly available due to their containing information that could compromise the privacy of research participants.

## Declaration of Generative AI and AI-assisted technologies in the writing process

No AI tools/services were used during the preparation of this work.
